# Case Report: Coronary-Pulmonary Fistula Closure by Percutaneous Approach: Learning From Mistakes

**DOI:** 10.3389/fcvm.2021.779716

**Published:** 2022-01-25

**Authors:** Vladimir Rubimbura, Grégoire Girod, Alain Delabays, David Meier, David C. Rotzinger, Olivier Muller, Salah D. Qanadli, Éric Eeckhout

**Affiliations:** ^1^Cardiology Department, Lausanne University Hospital, Lausanne, Switzerland; ^2^Cardiology Unit, Ensemble Hospitalier de la Côte, Morges, Switzerland; ^3^Cardiology Department, Sion Hospital, Sion, Switzerland; ^4^Radiology Department, Lausanne University Hospital, Lausanne, Switzerland

**Keywords:** fistula (coronary artery), percutaneous coronary intervention, shunt, dyspnea, congenital heart

## Abstract

Coronary-pulmonary artery fistulas (CPAF) are congenital vascular anomalies detected incidentally in most cases. When a significant left-right shunt exists, surgical, or percutaneous treatment is indicated. We describe a challenging case of CPAF closure, by percutaneous approach, in a patient symptomatic for dyspnea and evidence of a significant left-right shunt. A first attempt to close the fistula was performed implanting a vascular plug but it quickly embolized. The plug was successfully retrieved. In a second attempt, we deployed several coils before implanting the vascular plug with total closure of the fistula. The combination of plugs and coils is associated with a higher success rate of closure.

## Case Description

We describe the case of a 64-year-old patient admitted to our hospital for retrosternal chest pain. The patient presented progressive dyspnea over the last 5 years with a current the New York Heart Association (NYHA) stage III. Clinical examination was globally normal and did not reveal overt signs of heart failure.

The medical history of patient was unremarkable with no traditional cardiac risk factors.

**Figure 1:**
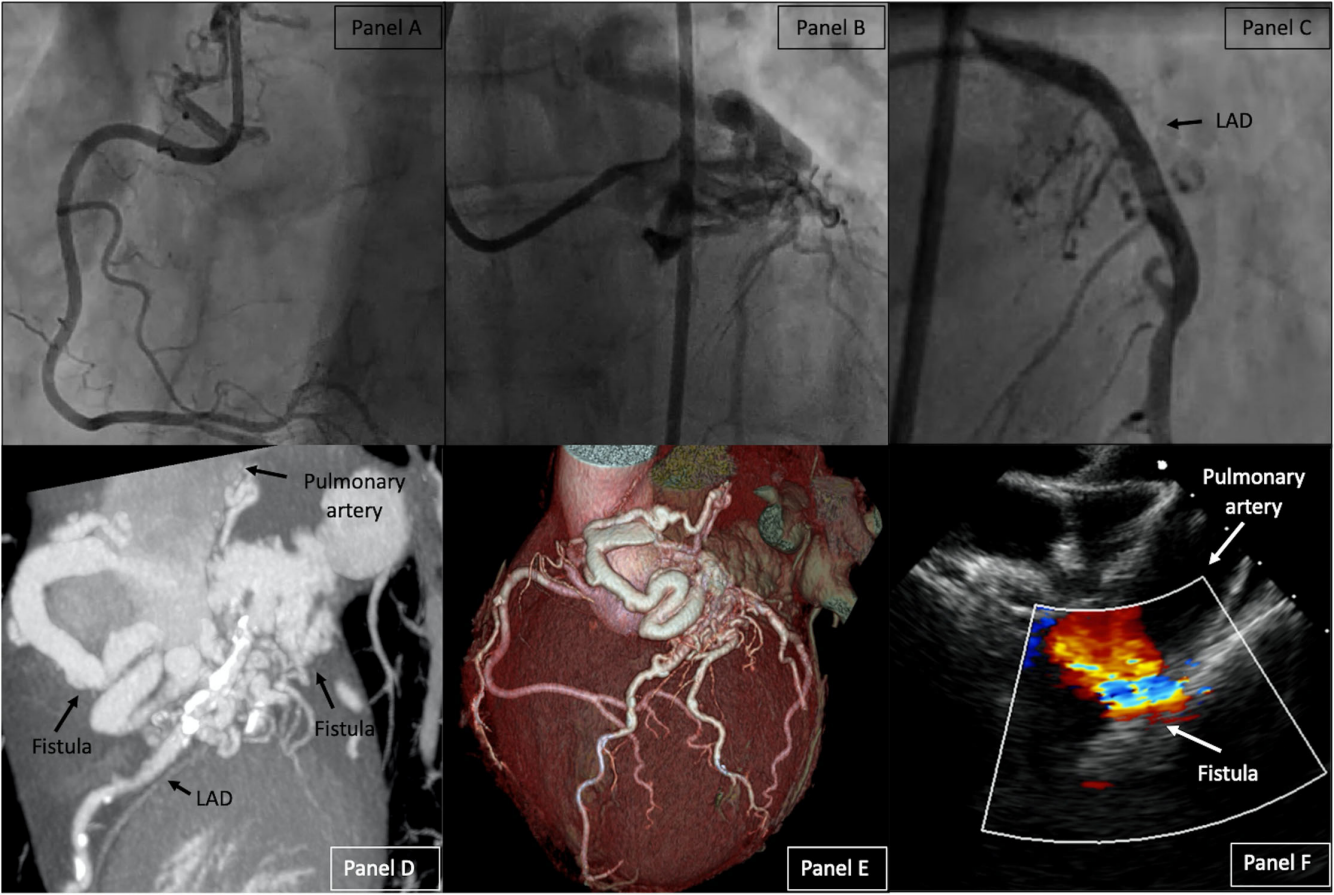
Right Coronary Artery (RCA) with the right part of the fistula **(A)**, Left Coronary Artery (LCA) with the left part of the fistula **(B)**, Selective LAD injection **(C)**. Baseline CCT with 2D Maximum intensity projection of the fistula **(D)** and 3D volume rendering of the heart **(E)**. TOE before fistula closure **(F)**. LAD, left anterior descending artery; CCT, *cardiac CT*; TOE, *transoesophageal echocardiogram*.

The ECG and transthoracic echocardiogram (TTE) were unremarkable. Laboratory tests were unremarkable. Coronary angiography revealed two fistulas arising from the right and left coronary arteries, both terminating in the pulmonary artery (PA) without significant stenosis visualized (Figures 1A,B; Supplementary Videos 1, 3). The left anterior descending artery (LAD) was injected selectively as it was poorly visualized behind the fistula (Figure 1C, Supplementary Video 2). Right heart catheterization (RHC) confirmed a significant left-to-right (L-R) shunt (Qp/Qs 1.6). Pulmonary function tests resulted in normal. A cardiac CT (CCT) scanner was performed, confirming a common merging of the two fistulas (6 mm of diameter) before entering the anterolateral left part of the trunk of the PA (Figures 1D,E).

After a heart-team discussion, a decision to close the fistulas with a percutaneous retrograde approach was taken.

The first attempt of closure was performed under local anesthesia. A Simmons (SIM I) catheter (Merit Medical, South Jordan, UT, USA) was used to cannulate the fistula, and an Amplatzer Vascular Plug IV 13.5/8 mm (Abbott, Chicago, IL, USA) was implanted with initial success (Supplementary Video 4). However, after 5 min, the device was embolized in the lower right PA and was successfully retrieved using an endovascular snare system (En-Snare, Merit Medical) (Supplementary Video 5). After discussion with the patient, a second percutaneous attempt was planned before considering surgical correction.

**Figure 2:**
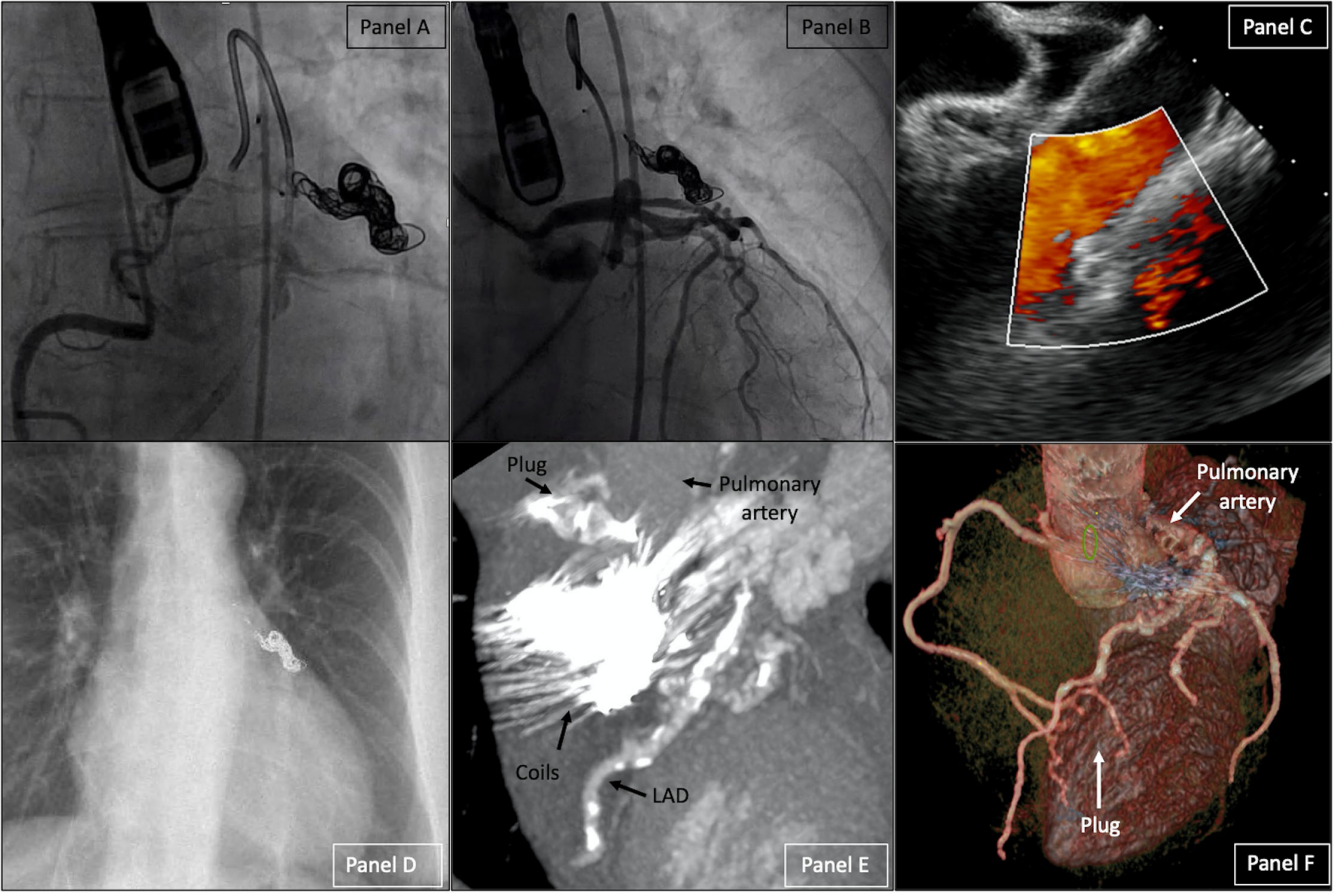
Right Coronary Artery (RCA) angiogram **(A)**, Left Coronary Artery (LCA) angiogram **(B)**, TOE **(C)**, chest radiography **(D)** after fistula closure. CCT after the intervention with 2D Maximum intensity projection of the fistula **(E)** and 3D volume rendering of the heart **(F)**. TOE, *transesophageal echocardiogram;* CCT, *cardiac CT*.

The second procedure was performed 3 months later under general anesthesia with the support of transesophageal echocardiography (TOE). Baseline TOE was performed (Figures 1E,F, Supplementary Video 6). The fistula was again cannulated by retrograde approach with the same SIM 1 diagnostic catheter. A microcatheter (Progreat, Terumo, Tokyo, Japan) was advanced over a 0.018” Glidewire Advantage (Terumo, Japan) distally in the fistula. Six detachable hydrocoils AZUR (Terumo, Japan), ranging from 10 to 14 mm in diameter and from 100 to 340 mm in length, were implanted (Supplementary Video 7). In the proximal part of the fistula, a plug AVP IV 13.5/8 mm (Abbott, USA) was implanted with a nearly complete closure of the fistula (Figures 2A–C; Supplementary Video 2, Supplementary Videos 8, 9). The post-operative care was uneventful with normal ECG and TTE controls. Thorax radiography confirmed the position of the plug close to the coils (Figure 2D). The patient was discharged on day 3.

The follow-up performed 3 months later revealed improvement in the symptoms without residual dyspnea. A control CCT confirmed the fistula's closure (Figures 2E,F).

## Discussion

Overall, coronary artery anomalies are rare in the general population. The most common coronary-artery fistulas (CAF) are coronary-pulmonary fistulas (CPAF) arising in most cases from both the right and left coronary arteries and terminating mostly in the main trunk of the PA (incidence ranging from 0.05 to 0.80%) (1, 2).

Coronary-pulmonary artery fistulas are incidental findings in most cases, but chest pain and dyspnea are the main findings when symptomatic (3). When a significant hemodynamic L-R shunt is present, surgical or percutaneous correction (coils, vascular plug occluders, covered stents, or a combination of different systems) is indicated and the choice depends on the technical feasibility of the latter (4, 5).

## Conclusions

Coronary-pulmonary artery fistulas are incidental findings in most cases and closure is indicated when symptoms are present and/or a significant left-to-right shunt exists. Percutaneous closure of the fistula is feasible after careful evaluation with multimodality imaging. The combination of plugs and coils is associated with a higher success rate of closure.

## Data Availability Statement

The original contributions presented in the study are included in the article/Supplementary Material, further inquiries can be directed to the corresponding author/s.

## Ethics Statement

Written informed consent was obtained from the participant for the publication of this case report.

## Author Contributions

VR, ÉE, and SQ designed and wrote the paper. GG, AD, DM, DR, and OM critically revised the paper. All authors contributed to the article and approved the submitted version.

## Conflict of Interest

The authors declare that the research was conducted in the absence of any commercial or financial relationships that could be construed as a potential conflict of interest.

## Publisher's Note

All claims expressed in this article are solely those of the authors and do not necessarily represent those of their affiliated organizations, or those of the publisher, the editors and the reviewers. Any product that may be evaluated in this article, or claim that may be made by its manufacturer, is not guaranteed or endorsed by the publisher.

## Supplementary Material

Supplementary Video 1 Left coronary angiogram.

Supplementary Video 2 Left coronary angiogram after implantation of the vascular plug and coils.

Supplementary Video 3 Right coronary angiogram.

Supplementary Video 4 Vascular plug deployment.

Supplementary Video 5 Retrieve the vascular plug from the right lower lobe pulmonary artery with an endovascular snare system.

Supplementary Video 6 Baseline transesophageal echocardiogram.

Supplementary Video 7 Coils release in the fistula.

Supplementary Video 8 Right coronary angiogram after implantation of the vascular plug and coils.

Supplementary Video 9 Final transesophageal echocardiogram.

## Abbreviations

CCT: Cardiac computed tomography
CAF: Coronary artery fistula
CPAF: Coronary-pulmonary artery fistula
ECG: Electrocardiogram
PA: Pulmonary artery
RHC: Right heart catheterization
TOE: Transesophageal echocardiogram
TTE: Transthoracic echocardiogram.
